# Pyridinium bis­(pyridine-κ*N*)tetra­kis­(thio­cyanato-κ*N*)ferrate(III)–pyrazine-2-carbo­nitrile–pyridine (1/4/1)

**DOI:** 10.1107/S1600536813010362

**Published:** 2013-04-20

**Authors:** Sergii I. Shylin, Il’ya A. Gural’skiy, Matti Haukka, Irina A. Golenya

**Affiliations:** a Department of Chemistry, Taras Shevchenko National University of Kyiv, Volodymyrska 64/13, 01601 Kyiv, Ukraine; b Department of Chemistry, University of Jyväskylä, PO Box 35, FI-40014 Jyväskyä, Finland

## Abstract

In the title compound, (C_5_H_6_N)[Fe(NCS)_4_(C_5_H_5_N)_2_]·4C_5_H_3_N_3_·C_5_H_5_N, the Fe^III^ ion is located on an inversion centre and is six-coordinated by four N atoms of the thio­cyanate ligands and two pyridine N atoms in a *trans* arrangement, forming a slightly distorted octa­hedral geometry. A half-occupied H atom attached to a pyridinium cation forms an N—H⋯N hydrogen bond with a centrosymmetrically-related pyridine unit. Four pyrazine-2-carbo­nitrile mol­ecules crystallize per complex anion. In the crystal, π–π stacking inter­actions are present [centroid–centroid distances = 3.6220 (9), 3.6930 (9), 3.5532 (9), 3.5803 (9) and 3.5458 (8) Å].

## Related literature

For the use of mol­ecular assemblies comprising cationic and anionic modules, see: Fritsky *et al.* (1998 ▶, 2004 ▶); Kanderal *et al.* (2005 ▶). For Fe^II^–thio­cyanate complexes with aromatic *N*-donor ligands indicating spin crossover, see: Gamez *et al.* (2009 ▶); Niel *et al.* (2001 ▶). For related structures, see: Moroz *et al.* (2010 ▶); Penkova *et al.* (2010 ▶); Petrusenko *et al.* (1997 ▶); Real *et al.* (1991 ▶).
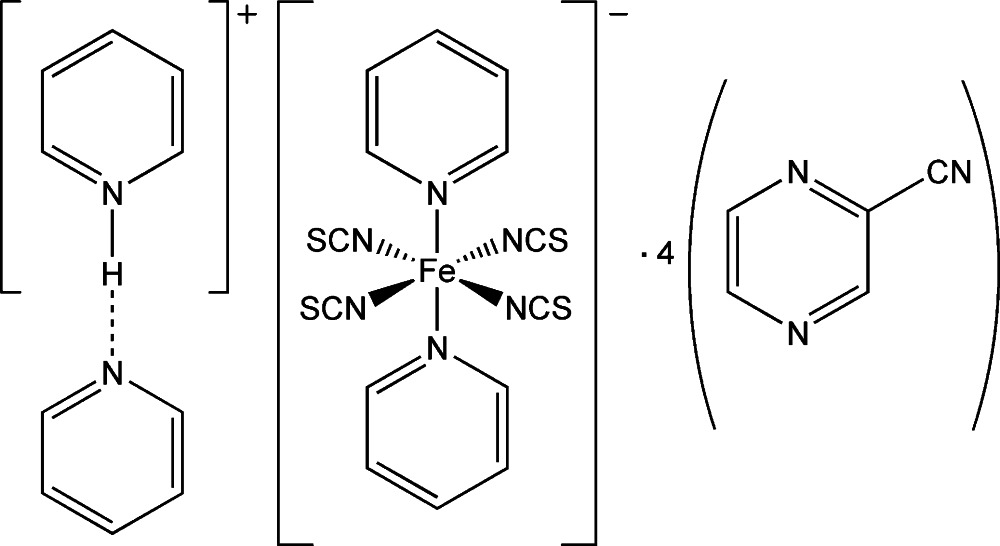


## Experimental

### 

#### Crystal data

(C_5_H_6_N)[Fe(NCS)_4_(C_5_H_5_N)_2_]·4C_5_H_3_N_3_·C_5_H_5_N
*M* *_r_* = 1025.99
Triclinic, 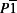

*a* = 8.1766 (2) Å
*b* = 11.9362 (3) Å
*c* = 12.7519 (3) Å
α = 102.982 (1)°
β = 97.799 (1)°
γ = 97.684 (1)°
*V* = 1184.02 (5) Å^3^
*Z* = 1
Mo *K*α radiation
μ = 0.55 mm^−1^
*T* = 120 K
0.38 × 0.19 × 0.17 mm

#### Data collection

Bruker Kappa APEXII DUO CCD diffractometer
Absorption correction: multi-scan (*SADABS*; Sheldrick, 1996 ▶) *T*_min_ = 0.818, *T*_max_ = 0.910
18588 measured reflections
5482 independent reflections
4470 reflections with *I* > 2σ(*I*)
*R*_int_ = 0.024

#### Refinement

*R*[*F* ^2^ > 2σ(*F* ^2^)] = 0.031
*wR*(*F* ^2^) = 0.076
*S* = 1.01
5482 reflections
313 parameters
H-atom parameters constrained
Δρ_max_ = 0.33 e Å^−3^
Δρ_min_ = −0.37 e Å^−3^

### 

Data collection: *APEX2* (Bruker, 2007 ▶); cell refinement: *SAINT* (Bruker, 2007 ▶); data reduction: *SAINT*; program(s) used to solve structure: *SHELXS97* (Sheldrick, 2008 ▶); program(s) used to refine structure: *SHELXL97* (Sheldrick, 2008 ▶); molecular graphics: *DIAMOND* (Brandenburg, 1997 ▶); software used to prepare material for publication: *SHELXL97*.

## Supplementary Material

Crystal structure: contains datablock(s) I, global. DOI: 10.1107/S1600536813010362/hy2622sup1.cif

Structure factors: contains datablock(s) I. DOI: 10.1107/S1600536813010362/hy2622Isup2.hkl

Supplementary material file. DOI: 10.1107/S1600536813010362/hy2622Isup3.cdx

Additional supplementary materials:  crystallographic information; 3D view; checkCIF report

## Figures and Tables

**Table 1:** Hydrogen-bond geometry (Å, °)

*D*—H⋯*A*	*D*—H	H⋯*A*	*D*⋯*A*	*D*—H⋯*A*
N4—H4*N*⋯N4^i^	0.88	1.80	2.677 (3)	179

Symmetry code: (i) 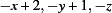.

**Fig. 1.**
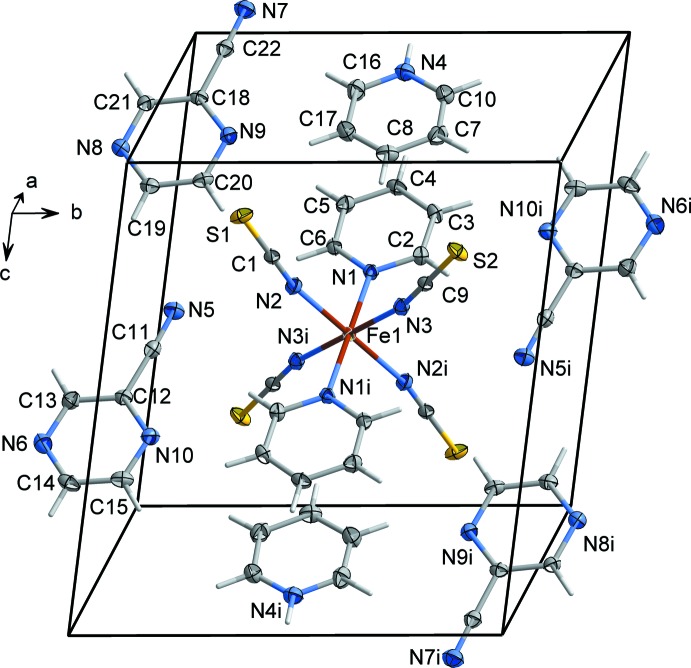
Molecular structure of the title compound with displacement ellipsoids drawn at the 50% probability level. [Symmetry code: (i) -x+1, -y+1, -z+1.]

**Fig. 2.**
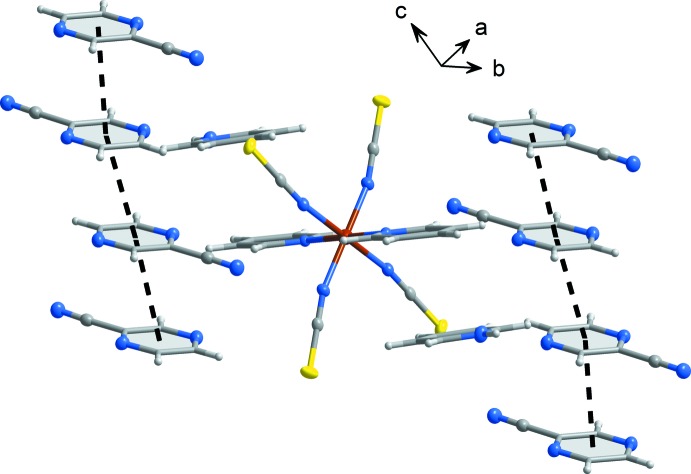
Crystal structure of the title compound, showing π–π contacts between the cnpz molecules as dashed lines (carmine: Fe, yellow: S, blue: N, grey: C, light-grey: H).

**Fig. 3.**
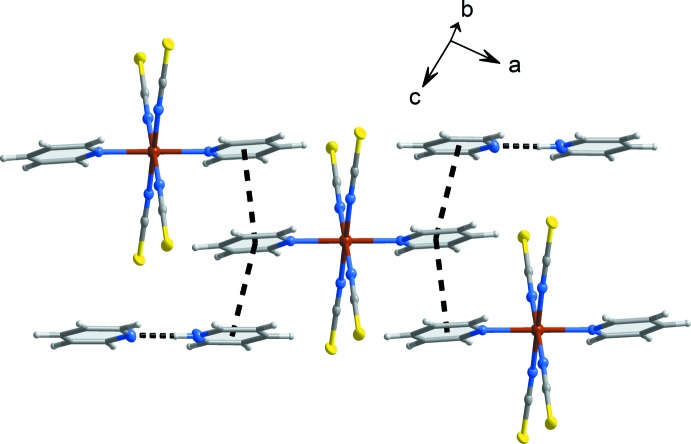
Crystal structure of the title compound, showing hydrogen bonds and π–π contacts between the pyridine molecules as dashed lines (carmine: Fe, yellow: S, blue: N, grey: C, light-grey: H).

## supplementary crystallographic information

### Comment

Molecular assemblies comprising from cationic and anionic modules are of special interest for crystal engineering and molecular magnetism (Fritsky *et al.*, 2004). Formation of such compounds often can be mediated by different types of intermolecular interactions, such as co-ordination and hydrogen bonds, ionic and van der Waals interactions (Fritsky *et al.*, 1998; Kanderal *et al.*, 2005). Such assemblies may possess interesting functional properties and, in particular, indicate spin crossover behavior. In this regard, Fe^II^ thiocyanate complexes with aromatic N-donor ligands attract much attention considering the possible metal ion spin state modulation by variation of a ligand (Gamez *et al.*, 2009) accompanied by coordination polymer formation. Particularly, as one of the simplest bridging N-donor ligands to design coordination polymers, pyrazine (pz) is known for the construction of spin crossover Hofmann-like clathrates with general formula [Fe^II^*M*^II^(pz)(CN)_4_]_∞_ (*M* = Ni, Pd or Pt) (Niel *et al.*, 2001). A combination of pz and thiocyanate ligands leads to the formation of two-dimensional coordination polymer [Fe(NCS)_2_(pz)_2_] with an antiferromagnetic exchange between metal centres (Real *et al.*, 1991). In this context, we attempted to synthesize Fe^II^ thiocyanate complex with pyrazine-2-carbonitrile (cnpz). However, the reaction of [Fe^II^(NCS)_2_(py)_4_] (py = pyridine) and cnpz in an organic media in air led to oxidation of Fe^II^ and to the formation of the title compound.

The compound consists of one complex anion [Fe(NCS)_4_(py)_2_]^-^, one pyridinium cation, one pyridine and four pyrazine-2-carbonitrile molecules (Fig. 1). The Fe^III^ ion is located on an inversion centre and is sixfold coordinated by four N atoms of four thiocyanate anions and two N atoms of two pyridine ligands in a *trans* arrangement, forming a slightly distorted octahedral coordination geometry. The thiocyanate ligands are bound through N atoms and are quasi-linear [S1—C1—N2 = 179.41 (14), S2—C9—N3 = 179.33 (15)°], while the Fe—NCS linkages are bent [Fe1—N2—C1 = 163.20 (12), Fe1—N3—C9 = 167.56 (12)°]. These structural features are typical for the complexes where the NCS group is N-bound (Petrusenko *et al.*, 1997). The distances between Fe^III^ ion and N atoms of the thiocyanate anions [Fe1—N2 = 2.0424 (13), Fe1—N3 = 2.0370 (13) Å] are considerably shorter than those between Fe^III^ and N atoms of the pyridine ligands [Fe1—N1 = 2.1320 (12)Å], that could be related to the higher affinity of the metal ion to negatively charged thiocyanate comparing with the neutral organic ligand. The C—N and C—C bond lenths in the coordinated pyridine ligands are normal and close to the values observed in the related structures (Moroz *et al.*, 2010; Penkova *et al.*, 2010).

In the title compound there are four solvent molecules of pyrazine-2-carbonitrile per each Fe^III^ ion that interact with one another through π–π stacking, with distances between the centroids of 3.5532 (9), 3.5803 (9) and 3.5458 (8) Å (Fig. 2). One of the uncoordinated pyridines is protonated and the N-bound H atom is disodered between two equally populated positions, forming N—H···N hydrogen bonds (Table 1). Coordinated and solvent pyridine molecules also interact with one another *via*π–π contacts, with distances between the centroids of 3.6220 (9) and 3.6930 (9) Å (Fig. 3).

### Experimental

Crystals of the title compound were obtained by adding pyrazine-2-carbonitrile (52.5 mg, 0.5 mmol) to tetrakis(pyridine)bis(isothiocyanato)iron(II), [Fe(NCS)_2_(py)_4_], (48.8 mg, 0.1 mmol) in acetone (5 ml). The solution was left to evaporate in air. In one day this yielded red crystals that were collected, washed with water and dried in air (yield: 21 mg, 20%).

### Refinement

H atoms were positioned geometrically and refined as riding atoms, with C—H = 0.95 and N—H = 0.88 Å and with *U*_iso_(H) = 1.2*U*_eq_(C,N). N-bound H atom of the uncoordinated pyridine is half-occupied due to the requirement of symmetry.

### Crystal data

**Table:**

(C_5_H_6_N)[Fe(NCS)_4_(C_5_H_5_N)_2_]·4C_5_H_3_N_3_·C_5_H_5_N	*Z* = 1
*M**_r_* = 1025.99	*F*(000) = 527
Triclinic, *P*1	*D*_x_ = 1.439 Mg m^−^^3^
Hall symbol: -P 1	Mo *K*α radiation, λ = 0.71073 Å
*a* = 8.1766 (2) Å	Cell parameters from 7810 reflections
*b* = 11.9362 (3) Å	θ = 2.6–27.6°
*c* = 12.7519 (3) Å	µ = 0.55 mm^−^^1^
α = 102.982 (1)°	*T* = 120 K
β = 97.799 (1)°	Block, red
γ = 97.684 (1)°	0.38 × 0.19 × 0.17 mm
*V* = 1184.02 (5) Å^3^	

### Data collection

**Table:**

Bruker Kappa APEXII DUO CCD diffractometer	5482 independent reflections
Radiation source: fine-focus sealed tube	4470 reflections with *I* > 2σ(*I*)
Curved graphite crystal monochromator	*R*_int_ = 0.024
Detector resolution: 16 pixels mm^-1^	θ_max_ = 27.7°, θ_min_ = 1.7°
φ and ω scans with κ offset	*h* = −10→10
Absorption correction: multi-scan (*SADABS*; Sheldrick, 1996)	*k* = −15→15
*T*_min_ = 0.818, *T*_max_ = 0.910	*l* = −16→16
18588 measured reflections	

### Refinement

**Table:**

Refinement on *F*^2^	Primary atom site location: structure-invariant direct methods
Least-squares matrix: full	Secondary atom site location: difference Fourier map
*R*[*F*^2^ > 2σ(*F*^2^)] = 0.031	Hydrogen site location: inferred from neighbouring sites
*wR*(*F*^2^) = 0.076	H-atom parameters constrained
*S* = 1.01	*w* = 1/[σ^2^(*F*_o_^2^) + (0.0363*P*)^2^ + 0.3715*P*] where *P* = (*F*_o_^2^ + 2*F*_c_^2^)/3
5482 reflections	(Δ/σ)_max_ = 0.001
313 parameters	Δρ_max_ = 0.33 e Å^−^^3^
0 restraints	Δρ_min_ = −0.37 e Å^−^^3^

### Special details

**Table:**

Experimental. Hydrogen atoms were positioned geometrically and constrained to ride on their parent atoms, with C—H = 0.95 Å, N—H = 0.88 Å, and *U*_iso_ = 1.2 *U*_eq_(parent atom). The highest peak is located 0.70 Å from atom C18 and the deepest hole is located 0.48 Å from atom Fe1.
Geometry. All e.s.d.'s (except the e.s.d. in the dihedral angle between two l.s. planes) are estimated using the full covariance matrix. The cell e.s.d.'s are taken into account individually in the estimation of e.s.d.'s in distances, angles and torsion angles; correlations between e.s.d.'s in cell parameters are only used when they are defined by crystal symmetry. An approximate (isotropic) treatment of cell e.s.d.'s is used for estimating e.s.d.'s involving l.s. planes.
Refinement. Refinement of *F*^2^ against ALL reflections. The weighted *R*-factor *wR* and goodness of fit *S* are based on *F*^2^, conventional *R*-factors *R* are based on *F*, with *F* set to zero for negative *F*^2^. The threshold expression of *F*^2^ > σ(*F*^2^) is used only for calculating *R*-factors(gt) *etc*. and is not relevant to the choice of reflections for refinement. *R*-factors based on *F*^2^ are statistically about twice as large as those based on *F*, and *R*- factors based on ALL data will be even larger.

### Fractional atomic coordinates and isotropic or equivalent isotropic displacement parameters (Å^2^)

**Table:**

	*x*	*y*	*z*	*U*_iso_*/*U*_eq_	Occ. (<1)
Fe1	0.5000	0.5000	0.5000	0.01474 (8)	
S1	0.18557 (5)	0.25687 (4)	0.16391 (3)	0.02717 (11)	
S2	0.31797 (5)	0.74869 (4)	0.27623 (4)	0.02695 (11)	
N1	0.72282 (15)	0.50545 (11)	0.43036 (10)	0.0163 (3)	
N2	0.38008 (16)	0.37328 (11)	0.36553 (11)	0.0200 (3)	
N3	0.42809 (15)	0.62208 (11)	0.42297 (11)	0.0193 (3)	
N4	0.86353 (18)	0.50989 (12)	0.04639 (11)	0.0279 (3)	
H4N	0.9528	0.5042	0.0153	0.033*	0.50
N5	0.40216 (18)	0.10177 (12)	0.42369 (12)	0.0278 (3)	
N6	0.09833 (17)	−0.12537 (12)	0.62053 (11)	0.0271 (3)	
N7	0.90967 (18)	0.11755 (13)	−0.07429 (12)	0.0302 (3)	
N8	0.59044 (17)	−0.09046 (12)	0.13024 (11)	0.0237 (3)	
N9	0.67103 (17)	0.14539 (11)	0.12764 (11)	0.0220 (3)	
N10	0.15882 (17)	0.11284 (12)	0.62270 (11)	0.0224 (3)	
C1	0.29957 (18)	0.32456 (13)	0.28113 (12)	0.0173 (3)	
C2	0.80428 (18)	0.60594 (13)	0.41827 (12)	0.0193 (3)	
H2	0.7656	0.6766	0.4459	0.023*	
C3	0.94253 (19)	0.60979 (14)	0.36697 (13)	0.0213 (3)	
H3	0.9981	0.6821	0.3597	0.026*	
C4	0.99889 (19)	0.50744 (14)	0.32645 (13)	0.0214 (3)	
H4	1.0925	0.5080	0.2897	0.026*	
C5	0.9167 (2)	0.40386 (14)	0.34019 (13)	0.0225 (3)	
H5	0.9541	0.3323	0.3142	0.027*	
C6	0.77991 (19)	0.40658 (13)	0.39215 (12)	0.0197 (3)	
H6	0.7235	0.3354	0.4014	0.024*	
C7	0.6803 (2)	0.62600 (15)	0.12971 (14)	0.0297 (4)	
H7	0.6508	0.7007	0.1539	0.036*	
C8	0.5837 (2)	0.52690 (15)	0.14229 (13)	0.0271 (4)	
H8	0.4869	0.5328	0.1754	0.032*	
C9	0.38283 (18)	0.67553 (13)	0.36185 (12)	0.0176 (3)	
C10	0.8197 (2)	0.61399 (15)	0.08152 (14)	0.0283 (4)	
H10	0.8867	0.6817	0.0730	0.034*	
C11	0.32510 (19)	0.07281 (13)	0.48410 (13)	0.0204 (3)	
C12	0.22339 (18)	0.03216 (13)	0.55739 (12)	0.0183 (3)	
C13	0.1944 (2)	−0.08487 (14)	0.55571 (13)	0.0238 (3)	
H13	0.2437	−0.1378	0.5074	0.029*	
C14	0.0340 (2)	−0.04577 (15)	0.68615 (13)	0.0255 (4)	
H14	−0.0350	−0.0704	0.7338	0.031*	
C15	0.0642 (2)	0.07162 (15)	0.68746 (13)	0.0252 (4)	
H15	0.0157	0.1246	0.7363	0.030*	
C16	0.7703 (2)	0.41443 (15)	0.05894 (15)	0.0309 (4)	
H16	0.8024	0.3407	0.0344	0.037*	
C17	0.6293 (2)	0.42005 (15)	0.10637 (14)	0.0292 (4)	
H17	0.5645	0.3511	0.1142	0.035*	
C18	0.72593 (18)	0.05927 (13)	0.06193 (12)	0.0182 (3)	
C19	0.53568 (19)	−0.00576 (14)	0.19602 (12)	0.0209 (3)	
H19	0.4667	−0.0256	0.2456	0.025*	
C20	0.5757 (2)	0.11054 (14)	0.19485 (12)	0.0221 (3)	
H20	0.5334	0.1675	0.2439	0.026*	
C21	0.68690 (19)	−0.05677 (14)	0.06260 (12)	0.0211 (3)	
H21	0.7296	−0.1138	0.0139	0.025*	
C22	0.82945 (19)	0.09294 (14)	−0.01369 (13)	0.0219 (3)	

### Atomic displacement parameters (Å^2^)

**Table:**

	*U*^11^	*U*^22^	*U*^33^	*U*^12^	*U*^13^	*U*^23^
Fe1	0.01377 (15)	0.01447 (15)	0.01549 (15)	0.00130 (11)	0.00307 (11)	0.00305 (12)
S1	0.0231 (2)	0.0345 (2)	0.0178 (2)	0.00335 (17)	0.00294 (16)	−0.00485 (17)
S2	0.0290 (2)	0.0289 (2)	0.0305 (2)	0.00821 (18)	0.00977 (18)	0.01836 (18)
N1	0.0150 (6)	0.0167 (6)	0.0168 (6)	0.0016 (5)	0.0026 (5)	0.0042 (5)
N2	0.0187 (6)	0.0182 (7)	0.0213 (7)	0.0017 (5)	0.0032 (5)	0.0018 (5)
N3	0.0177 (6)	0.0178 (7)	0.0224 (7)	0.0020 (5)	0.0045 (5)	0.0050 (5)
N4	0.0315 (8)	0.0282 (8)	0.0275 (8)	0.0064 (6)	0.0127 (6)	0.0086 (6)
N5	0.0284 (8)	0.0291 (8)	0.0268 (7)	0.0039 (6)	0.0103 (6)	0.0062 (6)
N6	0.0268 (8)	0.0264 (8)	0.0279 (8)	−0.0001 (6)	0.0022 (6)	0.0108 (6)
N7	0.0279 (8)	0.0364 (9)	0.0255 (7)	−0.0008 (6)	0.0082 (6)	0.0076 (6)
N8	0.0275 (7)	0.0216 (7)	0.0223 (7)	0.0020 (6)	0.0076 (6)	0.0054 (6)
N9	0.0244 (7)	0.0212 (7)	0.0212 (7)	0.0048 (5)	0.0052 (6)	0.0054 (6)
N10	0.0244 (7)	0.0238 (7)	0.0204 (7)	0.0061 (6)	0.0061 (5)	0.0059 (6)
C1	0.0160 (7)	0.0161 (7)	0.0215 (8)	0.0043 (6)	0.0085 (6)	0.0041 (6)
C2	0.0181 (8)	0.0165 (7)	0.0228 (8)	0.0018 (6)	0.0025 (6)	0.0048 (6)
C3	0.0174 (8)	0.0209 (8)	0.0259 (8)	−0.0004 (6)	0.0037 (6)	0.0083 (7)
C4	0.0145 (7)	0.0285 (9)	0.0223 (8)	0.0026 (6)	0.0059 (6)	0.0076 (7)
C5	0.0210 (8)	0.0217 (8)	0.0257 (8)	0.0070 (6)	0.0064 (7)	0.0045 (7)
C6	0.0206 (8)	0.0166 (8)	0.0219 (8)	0.0018 (6)	0.0045 (6)	0.0054 (6)
C7	0.0383 (10)	0.0239 (9)	0.0268 (9)	0.0102 (8)	0.0048 (8)	0.0038 (7)
C8	0.0264 (9)	0.0330 (10)	0.0236 (8)	0.0089 (7)	0.0064 (7)	0.0073 (7)
C9	0.0144 (7)	0.0164 (7)	0.0219 (8)	0.0011 (6)	0.0081 (6)	0.0023 (6)
C10	0.0363 (10)	0.0226 (9)	0.0265 (9)	0.0023 (7)	0.0065 (7)	0.0081 (7)
C11	0.0201 (8)	0.0194 (8)	0.0204 (8)	0.0036 (6)	0.0017 (6)	0.0029 (6)
C12	0.0158 (7)	0.0227 (8)	0.0158 (7)	0.0024 (6)	0.0007 (6)	0.0054 (6)
C13	0.0231 (8)	0.0232 (8)	0.0238 (8)	0.0032 (7)	0.0029 (7)	0.0042 (7)
C14	0.0191 (8)	0.0380 (10)	0.0202 (8)	0.0004 (7)	0.0017 (6)	0.0124 (7)
C15	0.0238 (8)	0.0337 (10)	0.0202 (8)	0.0080 (7)	0.0072 (7)	0.0073 (7)
C16	0.0364 (10)	0.0219 (9)	0.0371 (10)	0.0087 (7)	0.0108 (8)	0.0081 (8)
C17	0.0307 (9)	0.0255 (9)	0.0337 (10)	0.0032 (7)	0.0079 (8)	0.0119 (8)
C18	0.0153 (7)	0.0238 (8)	0.0149 (7)	0.0019 (6)	0.0016 (6)	0.0049 (6)
C19	0.0179 (8)	0.0277 (9)	0.0168 (7)	0.0021 (6)	0.0038 (6)	0.0055 (6)
C20	0.0228 (8)	0.0263 (9)	0.0178 (8)	0.0085 (7)	0.0056 (6)	0.0028 (6)
C21	0.0231 (8)	0.0211 (8)	0.0187 (8)	0.0045 (6)	0.0056 (6)	0.0021 (6)
C22	0.0207 (8)	0.0235 (8)	0.0197 (8)	0.0014 (6)	0.0025 (6)	0.0039 (6)

### Geometric parameters (Å, º)

**Table:**

Fe1—N3	2.0370 (13)	C6—H6	0.9500
Fe1—N2	2.0424 (13)	C7—C10	1.375 (2)
Fe1—N1	2.1320 (12)	C7—C8	1.385 (2)
S1—C1	1.6229 (16)	C7—H7	0.9500
S2—C9	1.6220 (16)	C8—C17	1.374 (2)
N1—C6	1.3412 (19)	C8—H8	0.9500
N1—C2	1.3430 (19)	C10—H10	0.9500
N2—C1	1.164 (2)	C11—C12	1.452 (2)
N3—C9	1.166 (2)	C12—N10	1.340 (2)
N4—C10	1.336 (2)	C12—C13	1.380 (2)
N4—C16	1.337 (2)	C13—H13	0.9500
N4—H4N	0.8800	C14—C15	1.386 (2)
N5—C11	1.142 (2)	C14—H14	0.9500
N6—C14	1.332 (2)	C15—N10	1.333 (2)
N6—C13	1.337 (2)	C15—H15	0.9500
N7—C22	1.141 (2)	C16—C17	1.375 (2)
N8—C19	1.330 (2)	C16—H16	0.9500
N8—C21	1.3355 (19)	C17—H17	0.9500
C2—C3	1.381 (2)	C18—N9	1.3411 (19)
C2—H2	0.9500	C19—C20	1.387 (2)
C3—C4	1.380 (2)	C19—H19	0.9500
C3—H3	0.9500	C20—N9	1.331 (2)
C4—C5	1.385 (2)	C20—H20	0.9500
C4—H4	0.9500	C21—C18	1.381 (2)
C5—C6	1.376 (2)	C21—H21	0.9500
C5—H5	0.9500	C22—C18	1.453 (2)
			
N3^i^—Fe1—N3	179.999 (1)	N1—C6—H6	118.6
N3^i^—Fe1—N2^i^	88.77 (5)	C5—C6—H6	118.6
N3—Fe1—N2^i^	91.23 (5)	C10—C7—C8	118.63 (16)
N3^i^—Fe1—N2	91.23 (5)	C10—C7—H7	120.7
N3—Fe1—N2	88.77 (5)	C8—C7—H7	120.7
N2^i^—Fe1—N2	180.0	C17—C8—C7	119.32 (16)
N3^i^—Fe1—N1	90.30 (5)	C17—C8—H8	120.3
N3—Fe1—N1	89.70 (5)	C7—C8—H8	120.3
N2^i^—Fe1—N1	90.58 (5)	N3—C9—S2	179.33 (15)
N2—Fe1—N1	89.42 (5)	N4—C10—C7	121.91 (16)
N3^i^—Fe1—N1^i^	89.70 (5)	N4—C10—H10	119.0
N3—Fe1—N1^i^	90.30 (5)	C7—C10—H10	119.0
N2^i^—Fe1—N1^i^	89.42 (5)	N5—C11—C12	177.71 (17)
N2—Fe1—N1^i^	90.58 (5)	N10—C12—C13	123.29 (14)
N1—Fe1—N1^i^	180.0	N10—C12—C11	116.65 (14)
C6—N1—C2	118.34 (13)	C13—C12—C11	120.04 (14)
C6—N1—Fe1	120.20 (10)	N6—C13—C12	121.47 (15)
C2—N1—Fe1	121.37 (10)	N6—C13—H13	119.3
C1—N2—Fe1	163.20 (12)	C12—C13—H13	119.3
C9—N3—Fe1	167.56 (12)	N6—C14—C15	122.38 (15)
C10—N4—C16	119.32 (15)	N6—C14—H14	118.8
C10—N4—H4N	120.3	C15—C14—H14	118.8
C16—N4—H4N	120.3	N10—C15—C14	122.39 (15)
C14—N6—C13	115.74 (14)	N10—C15—H15	118.8
C19—N8—C21	115.86 (14)	C14—C15—H15	118.8
N2—C1—S1	179.41 (14)	C15—N10—C12	114.73 (14)
N7—C22—C18	178.82 (18)	N4—C16—C17	121.84 (16)
N8—C21—C18	121.37 (14)	N4—C16—H16	119.1
N8—C21—H21	119.3	C17—C16—H16	119.1
C18—C21—H21	119.3	C8—C17—C16	118.98 (16)
N1—C2—C3	122.04 (14)	C8—C17—H17	120.5
N1—C2—H2	119.0	C16—C17—H17	120.5
C3—C2—H2	119.0	N9—C18—C21	123.30 (14)
C4—C3—C2	119.23 (14)	N9—C18—C22	116.67 (14)
C4—C3—H3	120.4	C21—C18—C22	120.03 (14)
C2—C3—H3	120.4	N8—C19—C20	122.36 (14)
C3—C4—C5	118.89 (14)	N8—C19—H19	118.8
C3—C4—H4	120.6	C20—C19—H19	118.8
C5—C4—H4	120.6	N9—C20—C19	122.46 (14)
C6—C5—C4	118.72 (15)	N9—C20—H20	118.8
C6—C5—H5	120.6	C19—C20—H20	118.8
C4—C5—H5	120.6	C20—N9—C18	114.64 (13)
N1—C6—C5	122.77 (14)		

Symmetry code: (i) −*x*+1, −*y*+1, −*z*+1.

### Hydrogen-bond geometry (Å, º)

**Table:**

*D*—H···*A*	*D*—H	H···*A*	*D*···*A*	*D*—H···*A*
N4—H4*N*···N4^ii^	0.88	1.80	2.677 (3)	179

Symmetry code: (ii) −*x*+2, −*y*+1, −*z*.

## References

[bb1] Brandenburg, K. (1997). *DIAMOND* Crystal Impact GbR, Bonn, Germany.

[bb2] Bruker (2007). *APEX2* and *SAINT* Bruker AXS Inc., Madison, Wisconsin, USA.

[bb3] Fritsky, I. O., Kozłowski, H., Sadler, P. J., Yefetova, O. P., Świątek-Kozłowska, J., Kalibabchuk, V. A. & Głowiak, T. (1998). *J. Chem. Soc. Dalton Trans.* pp. 3269–3274.

[bb4] Fritsky, I. O., Świątek-Kozłowska, J., Dobosz, A., Sliva, T. Y. & Dudarenko, N. M. (2004). *Inorg. Chim. Acta*, **357**, 3746–3752.

[bb5] Gamez, P., Costa, J. S., Quesada, M. & Aromí, G. (2009). *Dalton Trans.* pp. 7845–7853. 10.1039/b908208e 19771343

[bb6] Kanderal, O. M., Kozłowski, H., Dobosz, A., Świątek-Kozłowska, J., Meyer, F. & Fritsky, I. O. (2005). *Dalton Trans.* pp. 1428–1437. 10.1039/b418598f 15824781

[bb7] Moroz, Y. S., Szyrweil, L., Demeshko, S., Kozłowski, H., Meyer, F. & Fritsky, I. O. (2010). *Inorg. Chem.* **49**, 4750–4752. 10.1021/ic100555s 20441208

[bb8] Niel, V., Martinez-Agudo, J. M., Muñoz, M. C., Gaspar, A. B. & Real, J. A. (2001). *Inorg. Chem.* **40**, 3838–3839. 10.1021/ic010259y 11466039

[bb9] Penkova, L., Demeshko, S., Pavlenko, V. A., Dechert, S., Meyer, F. & Fritsky, I. O. (2010). *Inorg. Chim. Acta*, **363**, 3036–3040.

[bb10] Petrusenko, S. R., Kokozay, V. N. & Fritsky, I. O. (1997). *Polyhedron*, **16**, 267–274.

[bb11] Real, J. A., Munno, G., Muñoz, M. C. & Julve, M. (1991). *Inorg. Chem.* **30**, 2701–2704.

[bb12] Sheldrick, G. M. (1996). *SADABS* University of Göttingen, Germany.

[bb13] Sheldrick, G. M. (2008). *Acta Cryst.* A**64**, 112–122. 10.1107/S0108767307043930 18156677

